# A reference set of curated biomedical data and metadata from clinical case reports

**DOI:** 10.1038/sdata.2018.258

**Published:** 2018-11-20

**Authors:** J. Harry Caufield, Yijiang Zhou, Anders O. Garlid, Shaun P. Setty, David A. Liem, Quan Cao, Jessica M. Lee, Sanjana Murali, Sarah Spendlove, Wei Wang, Li Zhang, Yizhou Sun, Alex Bui, Henning Hermjakob, Karol E. Watson, Peipei Ping

**Affiliations:** 1 The NIH BD2K Center of Excellence in Biomedical Computing, University of California at Los Angeles, Los Angeles, CA 90095, USA; 2 Department of Physiology, University of California at Los Angeles, Los Angeles, CA 90095, USA; 3 Department of Cardiology, First Affiliated Hospital, Zhejiang University School of Medicine, 310003, Hangzhou, Zhejiang, P.R. China; 4 Department of Pediatric and Adult Congenital Cardiac Surgery, Miller Children’s and Women’s Hospital and Long Beach Memorial Hospital, Long Beach, CA 90806, USA; 5 Department of Medicine/Cardiology, University of California at Los Angeles, Los Angeles, CA 90095, USA; 6 Department of Bioinformatics, University of California at Los Angeles, Los Angeles, CA 90095, USA; 7 Department of Computer Science, University of California at Los Angeles, Los Angeles, CA 90095, USA; 8 Scalable Analytics Institute (ScAi), University of California at Los Angeles, Los Angeles, CA 90095, USA; 9 Department of Radiological Sciences, University of California at Los Angeles, Los Angeles, CA 90095, USA; 10 Molecular Systems Cluster, European Molecular Biology Laboratory-European Bioinformatics Institute, Wellcome Genome Campus, Cambridge, UK

**Keywords:** Metabolic disorders, Health services, Scientific data, Information technology, Education

## Abstract

Clinical case reports (CCRs) provide an important means of sharing clinical experiences about atypical disease phenotypes and new therapies. However, published case reports contain largely unstructured and heterogeneous clinical data, posing a challenge to mining relevant information. Current indexing approaches generally concern document-level features and have not been specifically designed for CCRs. To address this disparity, we developed a standardized metadata template and identified text corresponding to medical concepts within 3,100 curated CCRs spanning 15 disease groups and more than 750 reports of rare diseases. We also prepared a subset of metadata on reports on selected mitochondrial diseases and assigned ICD-10 diagnostic codes to each. The resulting resource, Metadata Acquired from Clinical Case Reports (MACCRs), contains text associated with high-level clinical concepts, including demographics, disease presentation, treatments, and outcomes for each report. Our template and MACCR set render CCRs more findable, accessible, interoperable, and reusable (FAIR) while serving as valuable resources for key user groups, including researchers, physician investigators, clinicians, data scientists, and those shaping government policies for clinical trials.

## Background and Summary

Clinical case reports (CCRs) are a fundamental means of sharing observations and insights in medicine. A wealth of knowledge exists within this venerated and actively growing area of medical publishing^1^. Unfortunately, many of these largely unstructured text data lack adequate metadata denoting specific clinical events. As a result, our ability to curate a comprehensive set of reports relevant to a disease of interest is inadequate, and extraction of the clinical insights contained within is limited. Our Metadata Acquired from CCRs (MACCRs), prepared by domain experts, enriches CCRs with detailed metadata, establishing a findable, accessible, interoperable, and reusable (FAIR)^2^ data resource and empowering researchers to form *in silico* cohorts of disease, amplify small sample size studies, and leverage the cumulative power of many reports for statistical analyses.

More than 1.89 million CCRs have been published as of August 2018, with over half a million in the last decade. CCRs serve as teaching tools, elucidating the reasoning behind diagnoses and management conclusions. Throughout history, CCRs have provided accounts of emerging diseases, their treatments, and their genetic backgrounds^3–5^. The first treatment of human rabies by Louis Pasteur in 1885^6,7^, the first application of penicillin in patients^8^, and an early study of the retroviral human T-cell lymphoma virus (HTLV)^9^ were all reported through CCRs^3^. CCRs are a first line of evidence, serving as both a source of individual pathologies and the basis of study for population-level trends that may otherwise go unnoticed^5,10^. These reports remain the only formally published mechanism for exchanging clinical observations and are not subject to the extensive privacy concerns of electronic health records (EHRs).

The text data in these CCRs are largely unstructured, vary widely in content, and contain interrelated phenomena, limiting their use as a structured resource. Existing resources for indexing and enforcing structure on biomedical documents, including Medical Subject Headings (MeSH) and their associated tools^11,12^, the ongoing Informatics for Integrating Biology and the Bedside (i2b2) curated resources^13,14^, and the CRAFT^15^, AnatEM^16^, NCBI disease^17^, and newly-available PubMed Phrases^18^ corpora are useful for focused natural language processing (NLP) approaches. Annotations of free-text resources, such as adverse reactions on drug labels^19^, are additional sources of expert-guided structure for unstructured text. However, these resources have limited applications with CCRs, as most of them have not been designed to model clinical narratives. As a result, there exists a significant gap between the clinical data we generate and our ability to convert it to knowledge. Clinical controlled vocabularies and coding systems (e.g., ICD-10^20^ or LOINC^21^) can help to bridge this gap but have rarely been used with published clinical reports. Furthermore, despite the existence of several resources (e.g., immuneXpresso^22^) offering utility on biomolecular text mining in the context of disease, information is fragmented with limited clinical perspective. Therefore, we set out to expand the value of CCRs as a vital biomedical knowledge resource through extensive metadata creation.

**Figure 1:**
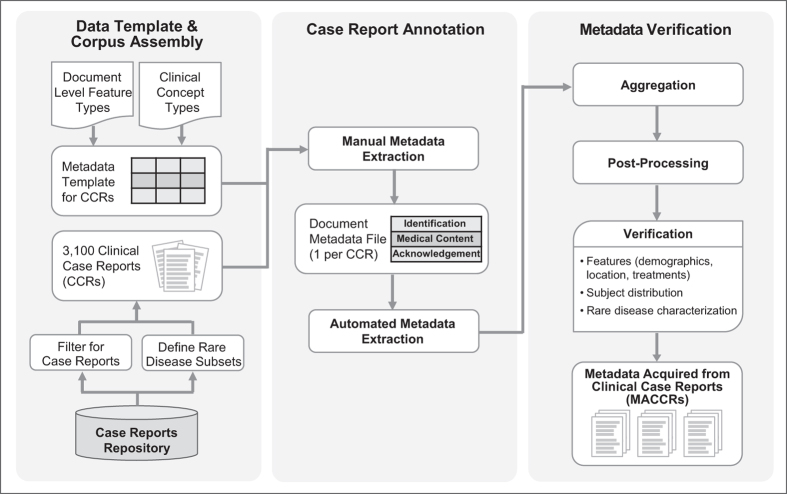
Data creation workflow. To assemble the set of Metadata Acquired from Clinical Case Reports (MACCRs) we first assembled a corpus of 3,100 published case reports. Using a document metadata template including document-level identification and acknowledgement features (i.e., citation data such as title; Medical Subject Headings [MeSH terms]) and concept-level medical content features (e.g., descriptions of patient demography, clinical signs and symptoms, or outcomes), a team of medical experts manually identified text from each document corresponding to each feature. More specific terms were identified through automated approaches. To finalize this dataset, we aggregated all document metadata records into a single file. We normalized categorical features, verified, and cleaned these data, which are available as the MACCR set.

**Figure 2:**
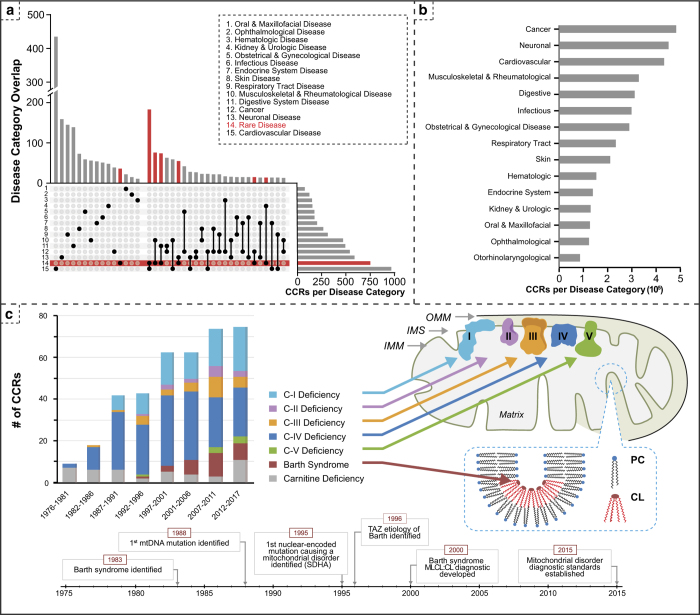
Contents of the MACCR dataset. (**a**) Concept overlap among CCRs in the MACCR set. We assigned each report to one or more disease categories based on involvement of particular organ systems. Reports describing presentations of rare diseases, including mitochondrial rare diseases, were assigned to the Rare Disease category as well. Here, we show the total count of reports labeled with single or multiple disease categories (Disease Category Overlap, top) as an UpSet plot^59^. Counts of reports involving rare diseases (n = 751) are highlighted in red. Total counts irrespective of overlap with other categories are also shown at right. For example, 435 CCRs involve cardiovascular disease (CVD) without specific involvement of other organ systems, yet 967 CCRs involve CVD alone *or* along with other disease categories. Otorhinolaryngologic reports constitute the smallest category in the MACCR set (n = 58); their counts are omitted here. (**b**) Distribution of disease categories across all published case reports. Here, we determined disease category assignment across all 1.89 million published CCRs (as of August 2018) using sets of MeSH terms corresponding to each category. As in Part A, a report may belong to multiple categories. More than a quarter of all reports in this broad set involve cancer, differing from the report distribution in the MACCR set, though in both sets, cancer, cardiovascular disease, and neuronal disease are the most common three disease categories. (**c**) Contribution of mitochondrial disease CCRs. 246 CCRs cover a sample of rare mitochondrial diseases, including Barth syndrome, carnitine deficiency, and deficiencies of the respiratory chain complexes. The distribution of CCR publication year is displayed on the left for each disease, and the affected components of the mitochondrion are represented in the diagram to the right. Complex I, II, III, IV, and V deficiencies each cause impairment in their respective component of the respiratory chain, resulting in a range of cardiovascular, neurological, muscular, and metabolic phenotypes. Barth syndrome is caused by a mutation in the tafazzin protein that renders it incapable of creating properly formed cardiolipin (CL) for the inner mitochondrial membrane (IMM). Phosphatidylcholine (PC) is unable to form the tight bends of the cristae, severely limiting energy generation and leading to cardiovascular complications. The timeline below depicts key advancements and discoveries relating to rare mitochondrial disease diagnosis. OMM: outer mitochondrial membrane. IMS: intermembrane space.

This MACCR dataset contains free-text selections from 3,100 CCRs, with over 32,000 manually annotated medical features across a variety of clinical presentations structured into 15 different types of medical concepts (Table 1; Figs 1 and 2). We curated the CCRs to ensure they would provide a general model of clinical language, then performed expert-guided annotation to extract a comprehensive and nuanced array of textual features. As CCRs often describe infrequently observed symptoms and diseases, our MACCR set includes over 700 reports of rare disease presentations, with additional focus on 7 selected mitochondrial diseases. Our goal in developing this resource is to provide a manageable, structured set of metadata on clinical events and case descriptions. In so doing, we render case reports more findable and the information contained within more accessible. One effect of making CCRs more FAIR is to facilitate the discovery and study of these invaluable sources of clinical insight, enhancing the potential for researchers and clinicians to gain a better understanding of diseases and their treatment. To guide those interested in employing the MACCR dataset in their research, we present 5 feasible primary study objectives (detailed in Usage Notes) that may be pursued through downstream analysis by researchers, physician investigators, clinicians, data scientists, IP officers, pharmaceutical companies, and those shaping government policies for clinical trials.

## Methods

To assemble our set of Metadata Acquired from Clinical Case Reports (MACCRs), we performed three primary stages of tasks. First, we designed a structured data template including the primary features of most case reports and curated a corpus of case report documents along with their associated metadata records. Next, we extracted metadata from each document in the corpus using manual and automated methods. Finally, all metadata records were aggregated into a single set of documents and verified. An overview of our approach is provided in Fig. 1.

### Metadata template and curation

Our data template (Table 1) includes metadata on three aspects of information collected for each CCR: identification, medical content, and acknowledgements. The identification features are primarily used to distinguish documents by bibliographic features, including the title, authors, journal, publication date, and unique identifiers such as DOI and PMID. The majority of the metadata collected in the medical content is concept-level. This includes text segments corresponding to 15 different clinical concepts, detailed in Table 1, where a segment may vary in length from a single word to multiple sentences. A single concept may also correspond to multiple text segments within a given document. The acknowledgements metadata provides details for disclosures and sponsorship.

The metadata in our dataset are sourced from CCRs indexed by MEDLINE. We first defined the CCR corpus from which our metadata are extracted using our *heartCases* software (https://github.com/UCLA-BD2K/heartCases); *heartCases* aggregates metadata provided with each MEDLINE entry to determine features common to a set of documents (e.g., their top publication years, journals, and MeSH descriptors). We then assigned each CCR to at least one of 16 disease categories based on their MeSH descriptors or presence of a MeSH descriptor in a title (Table 2). The set of terms for each disease category was defined using corresponding segments of the MeSH Tree (https://meshb.nlm.nih.gov/treeView); for example, all CCRs in the “digestive” category match primary and entry terms at or below the following points on the MeSH Tree: A03.159, A03.556, A03.620, A03.734, C06.130, C06.198, C06.267, C06.301, C06.405, C06.552, C06.689, C06.844, and G10.261, or a total of 3,356 different terms. A match may include a matched MeSH term used to index the document or presence of the term in the document title. With the goal of assembling a generally representative set of CCRs, we selected documents from the larger corpus such that the assignment to each disease category resembled that seen across all CCRs. The most popular topics across CCRs in general are therefore popular topics in our source CCR set as well. In an effort to ensure representation of a variety of disease presentations – one of the inherent strengths of case reports – we also selected reports of rare diseases (i.e., those affecting fewer than 200,000 individuals in the United States, as defined by the NIH NCATS Genetic and Rare Diseases Information Center [https://rarediseases.info.nih.gov/diseases/]) such that the set included 5 to 10 reports each for over 100 rare diseases. The resulting reference metadata set is sourced from 3,100 CCRs spanning 15 major disease categories, as well as a subset of rare diseases (Table 2).

Manual annotation was performed by 12 annotators familiar with medical terminology and, for subsets of rare diseases, with the clinical features underlying these diseases (e.g., for rare mitochondrial diseases, annotators possessed an understanding of the underlying mitochondrial physiology and corresponding mutations). Our roster of curators comes from clinical fellows (4), post-doctoral fellows (6), and senior graduate students (2). Full instructions followed by annotators are detailed in our *Metadata Extraction Guide*, included with the data files (Metadata Extraction Guide, Data Citation 1). For each CCR in the corpus, one annotator read the full text of the document and extracted text phrases corresponding to each component of the data template, avoiding extended discussion sections or clinical studies except when these sections were the only source of target metadata. They populated the template with these phrases, delimiting each distinct phrase with a semicolon, then completed the annotation set by adding bibliographic metadata as presented in the document. Annotators assigned each CCR one or more of 16 different disease categories based on both document content and MeSH descriptors, each corresponding to an organ system or general classification of disease presentations (e.g., cardiovascular, endocrine, infectious, or rare disease, etc.; Table 2). These categories were assigned based on presence of related symptoms, co-morbidities, and etiologies rather than primary diagnosis in order to determine conceptual overlap between cases. We did not include a category for congenital or genetic disorders and focused instead on the clinical signs of these presentations. Finally, annotators determined the count of additional data elements in each CCR, counting clinical images (i.e., any photograph, micrograph, or direct result of a diagnostic procedure such as an electrocardiogram), figures (i.e., any assembled image or data visualization), tables, and videos, not counting supplemental materials.

### ICD codes and interoperability

In order to facilitate interoperability with existing ontologies and support efforts at gaining a systematic understanding of disease, we assigned codes from a standard set of diagnostic identifiers to reports from a subset of the MACCR set. We sought to reveal shared and common symptoms as well as rare and unique characteristics underlying mitochondrial disease, constructing digital maps of disease symptomology for documents in the rare mitochondrial disease (RMD) subset using ICD-10-CM codes (International Statistical Classification of Diseases and Related Health Problems, tenth revision, clinical modification, 2018 release). The document corpus is assembled from 246 CCRs, each describing an individual presentation of one of six selected mitochondrial diseases (Barth syndrome, primary carnitine deficiency, or a deficiency of mitochondrial complex II, III, IV or V). For each document, two separate annotators familiar with mitochondrial disease pathologies identified specific concepts within the full text of the case report corresponding to ICD-10-CM codes, including those for symptoms. Codes are included if their concepts are part of a given patient’s clinical presentation, but not if they are only discussed or proposed. In final data files, observations are treated as binary values (1 if identified, 0 if not identified) and split into two different sets: in the first, each code is provided separately, while in the second, codes are aggregated into categories on the basis of their ICD-10 code blocks (https://www.cdc.gov/nchs/data/icd/10cmguidelines_fy2018_final.pdf). For example, one or more codes between C00 and D49 assigned for a given document will yield a score of 1 for the “Neoplasms” category corresponding to this code block. By incorporating ICD-10 codes and MeSH descriptors, we enhance interoperability in this subset of the MACCR set.

### Data quality control and validation

Quality control was implemented throughout the manual curation process with regular milestone meetings and a closely collaborative research environment designed to align and standardize methods for metadata extraction and foster consistency among all curators. Manual metadata extraction from individual CCRs was followed by aggregation and additional quality control through post-processing. All metadata records were combined into a single file using Python and R scripts (*Extract.py* and *QualityControl*.*R*; see Code Availability). This workflow uses basic natural language processing functions to perform the following: retrieval and verification of document details (e.g., title, database identifiers, and publication details), assignment of each document to one of our major disease categories, and conversion of patient age to a numerical value. For patient age identification in particular, all values are treated as integers, rounded down (e.g., a patient aged 5.5 years is assigned a value of 5; those aged <1 year are assigned a value of zero), or are estimated when not explicitly provided (e.g., a report of a patient in their “sixties” is assigned an age value of 65). Features with insufficient detail in the text are assigned a value of “NA”. All text features are checked to ensure formatting consistency, and to verify database cross-links: titles and author names are compared to MEDLINE records, DOIs and links are confirmed and added where missing, and journal names are normalized to include their full names as presented in the NLM Catalog (https://www.ncbi.nlm.nih.gov/nlmcatalog/journals), but with the preceding “the” omitted. All text field delimiters (i.e., those denoting separate text segments within a single field) are confirmed to be semicolons. Additionally, most text fields are converted to lowercase characters to enable easier comparison and named entity recognition (NER^23,24^). Final validation of the dataset is performed through observation of distributions of features within the MACCR set comparable to those seen in larger collections of clinical documents (see Technical Validation for additional details).

Geographic distribution analysis is performed for validation and visualization of MACCR set features. We developed an R Shiny app designed specifically for performing this analysis with clinical case reports (see Code availability). Briefly, this app first identifies the occurrence of all case reports indexed by MEDLINE on the basis of their AD, or Affiliation, field. This field is largely unstructured and has changed in format over time, so in order to provide additional detail and consistency, the text is processed to identify specific names of countries and US states. Then, the institutional affiliation field of each MACCR record is parsed in an identical manner. Using a 2-proportion Z-test, counts of locations are compared between the set of all available CCRs and those in the subset contributing metadata to our MACCR set to identify locations with a statistically significant (i.e., higher or lower) difference in proportion. The differences are visualized on a world map with US states treated independently from each other.

Three additional files contain citation details, corresponding MeSH headings, and named entities contained within the MACCR set. The citations for documents corresponding to each metadata record are loaded into a Mendeley citation database and converted to BibTeX format. The list of all unique MeSH headings is prepared by isolating unique MeSH descriptors, without modifiers, from each MEDLINE-format citation of those corresponding to each metadata record in the MACCR set. To determine the extent to which the metadata text segments correspond to a controlled vocabulary of biomedical terms (i.e., with NER), we identify all named entities up to three words long present in each medical content field across all 3,100 MACCRs, based on entities within MeSH and SNOMED-CT as per the UMLS Metathesaurus^25^.

### Code availability

The code used to process and verify the MACCR dataset, along with documentation, is available at https://github.com/UCLA-BD2K/MACCRs. This repository includes all utilities used in the assembly and verification of the MACCR metadata set, with the exception of the following two pieces of software. Analysis of the complete corpus of CCRs, as part of the verification of this dataset, was done using *heartCases*, available at https://github.com/UCLA-BD2K/heartCases. The R Shiny App used for analysis and visualization of case report geographic distributions is available at https://github.com/UCLA-BD2K/Significant-Mapping.

## Data Records

Starting from the manually curated set of CCRs as defined above, we obtain the full text records of each report from PubMed/MEDLINE corresponding to each respective PubMed entry identifier (PMID). Text corresponds to the PubMed Central document version where possible; all other text is curated from publicly-available document PDFs provided by journal publishers. As CCRs vary in structure and format (e.g., section headings vary, and a case description may be just one component of a document), curators identify a single, primary case presentation section within each published record, then identify text corresponding to the concepts within the CCR metadata extraction template (Table 1). The contents of the MACCR set are, therefore, metadata with respect to each CCR.

The primary data file (MACCRs.tsv, Data Citation 1) is provided in UTF-8, tab-delimited format, such that the metadata for each CCR is a single line in the file. Each column corresponds to a distinct metadata type. Within text columns, distinct text segments are delimited using semicolons and most are in lowercase only to facilitate easier searching. This file contains 1 metadata record for each of 3,100 unique documents. The corresponding reports have publication dates spanning from 1956 to 2018 and were originally published in 1,020 different journals. Across the 15 different concept-level free-text features identified within each report, the set contains 2,980 unique descriptions of diagnostic techniques/procedures and 3,026 unique clinical diagnoses, among other descriptions in context. Descriptions of all data fields are provided in our *MACCR File Guide* (MACCR File Guide, Data Citation 1). Full citations for all CCRs in the MACCR set are provided in BibTeX format within the citation file (MACCR_citations.bib, Data Citation 1).

We have provided our metadata extraction template (TEMPLATE.xlsx, Data Citation 1) to facilitate adaptation to new studies through the creation of similar CCR-based datasets. The template is provided as an Excel spreadsheet to ensure universality and ease of use. Each data type is identified in each row of the first column and corresponding values extracted from the CCR text are provided in the fourth column. The fifth column is used to identify values provided through PubMed records and indexing alone; for medical content, these values are MeSH descriptors (if they exist) and any provided modifiers. The second and third columns contain counts to indicate the presence of content in the fourth and fifth columns, such that a value of ‘1’ corresponds to any value other than a blank cell. To enable this comparison, cells without any relevant information for a particular CCR are left blank. Please see the *Metadata Extraction Guide* (Metadata Extraction Guide for the MACCR set, Data Citation 1) for further details of the process.

We additionally provide the set of all unique MeSH headings applicable to the source CCRs for the MACCR set (MACCR_mesh.tsv, Data Citation 1). This file provides a list of headings in the first column, with one unique heading per line and without further modifications. The second column provides the MeSH root category (as per the 2018 release; https://www.nlm.nih.gov/mesh/) for each heading as a single letter, e.g., the heading “Spleen” is in the Anatomy category, or category A. For headings with multiple potential codes within the MeSH hierarchy, the category of the first listed in the MeSH index file is used (e.g., “Outcome Assessment (Health Care)” has codes in categories H and N and is considered to be in category H). The headings “Male” and “Female” have no location in the MeSH tree and no category. Here, all reports contributing metadata to the MACCR set have at least one associated MeSH descriptor through MEDLINE.

Named entity recognition (NER)^23,24^ results (MACCR_entities.tsv, Data Citation 1) are provided as an additional means of illustrating concepts within the MACCR set. Each line in this file contains the PMID of the report corresponding to each metadata set, followed by a list of named entities identified within selected metadata fields, as indicated in the heading. Named entities are MeSH descriptors and SNOMED-CT terms, as available through UMLS resources^25^.

Mitochondrial disease reports, covering six different diseases (Barth syndrome, primary carnitine deficiency, or a deficiency of mitochondrial complex II, III, IV or V), contribute 246 reports to this dataset. As the majority of these reports describe rare mitochondrial diseases, we refer to this subset as the RMD subset. Metadata in the RMD subset include codes from ICD-10-CM such that symptoms and diagnoses mentioned within each CCR are each identified using the most closely matching and specific ICD-10-CM code, including symptom codes (codes R00.0 through R99). 500 unique codes were identified across all RMD CCRs, with a total of 2,119 codes assigned. The presence or absence of each of these 500 ICD-10-CM codes for each CCR is provided in its own file (MACCR_RMD_ICD10.tsv, Data Citation 1), with one CCR per row, identified by its PMID in the first column and the RMD category (barth [for Barth syndrome], carnitine [for primary carnitine deficiency], or complex_I through complex_V [for mitochondrial complex deficiencies]) in the second column. Each of the 500 unique ICD-10-CM codes identified in the RMD set is represented in the following 500 columns. A value of “1” indicates a given code matches clinical events described in the CCR, while a value of “0” indicates matching material is not observed, though its omission from CCR text may not conclusively indicate its absence. Additionally, we provide a compressed version of these data indicating presence or absence of categories of codes in lieu of individual codes (MACCR_RMD_ICD10_Categories.tsv, Data Citation 1). This file contains the PMID for one CCR and an RMD category in its first and second columns, respectively, as in the file described above. The following columns correspond to one of 20 chapter titles from ICD-10-CM, each represented by a block of codes. A value of “1” indicates at least one ICD-10-CM code in the specified chapter’s code block matches clinical events described in the CCR, while a value of “0” indicates matching material was not observed. Inclusion of category-based observation only in this file reduces the total observations to 1,073 across 20 blocks of codes.

This dataset itself meets the FAIR Data Principles. All files are provided through both Figshare (Data Citation 1) and through Dryad (Data Citation 2). Metadata are assigned a globally unique and persistent identifier and registered through both of these searchable resources to make them *F*indable; the metadata are retrievable via this identifier using an open, standardized, and free communications protocol to make them *A*ccessible; the metadata set uses a formal, broadly applicable vocabulary and domain-recognized ontologies (MeSH and ICD-10) to make them *I*nteroperable; and the metadata files contains detailed provenance, licensing, and versioning information to make them *R*eusable. Out of the 9 metrics used by FAIRShake (https://fairshake.cloud/), our dataset provides all 9 of the necessary values (Table 3).

## Technical Validation

### Distribution of disease categories *vs.* published case reports

We intend the MACCR set to be representative of the semantic and lexical variation present in reports from a wide variety of medical presentations and specialties. Rather than focusing on reports describing a single type of disease, the metadata in the MACCR set is sourced from clinical presentations spanning 15 disease groups, along with an additional category for rare disease presentations (Fig. 2a, and Table 2). CCRs often describe rare disease presentations and clinically-relevant relationships not accessible through any other public source. As one example, CCRs provided evidence for the theory that gadolinium-based contrast agents are a trigger for the rare disease nephrogenic systemic fibrosis^26,27^. By including a wide variety of medical concepts in our corpus, we highlight the value of CCRs, particularly in their descriptions of novel and often complex diagnostic processes. Further, we provide validation of MACCR content relative to that seen across all CCRs outside the corpus.

Both the MACCR set and the set of all published CCRs are predominantly composed of reports of cancer, cardiovascular disease, and neuronal disease (Fig. 2a-b). The variety of topics covered within the MACCR set is demonstrated by the number of MeSH descriptors among the contributing reports: the source reports for the MACCRs are indexed with 5,326 unique terms (MACCR_mesh.tsv, Data Citation 1), or 12,980 unique terms with their modifiers. For comparison, the full 2018 MeSH ontology includes 28,239 descriptors (https://www.nlm.nih.gov/mesh/) and the set of all 1.89 million CCRs published as of August 2018 is indexed with 24,842 unique terms, or 786,256 unique terms with modifiers. Of the 5,326 MeSH terms in the MACCR set, 2,042 describe Diseases (category C), 1,208 describe Chemicals and Drugs (category D), and 793 describe Analytical, Diagnostic and Therapeutic Techniques, and Equipment (category E).

### Rare disease subgroup membership

Our curation process ensures that the MACCR set includes descriptions of rare diseases across a variety of disease subtypes. Rare diseases are defined as those with an incidence in the U.S. of fewer than 1 in 20,000 individuals. These diseases are infrequently reported on in CCRs, primarily due to their inherent rarity. The contribution of these reports to the MACCR set offers a novel advantage by providing a more comprehensive representation of the language used in presentations of these diseases as compared to more frequently described clinical presentations. Figure 2a highlights the contribution of rare diseases to the MACCR set.

The MACCR set includes a focused subset of 246 rare mitochondrial disease (RMD) reports that describe presentations of one of six rare mitochondrial diseases. The distribution of these reports in the MACCR set across their years of publication reflects time points of discovery and increased diagnosis (Fig. 2c, left); the timeline presented in Fig. 2c highlights significant advances in the identification of mitochondrial diseases and their etiology, as well as the development of diagnostic tools and standards. These mitochondrial diseases in particular can be distinguished on the basis of deficiencies in crucial molecular components: complex I, II, III, IV, and V deficiencies impair individual components of the respiratory chain, while Barth syndrome is characterized by malformed cardiolipin, disturbing mitochondrial membranes and reducing respiratory chain function (Fig. 2c, right). Though the resulting sets of symptoms overlap noticeably, the overall range of symptoms covers cardiovascular, neurological, muscular, and metabolic phenotypes, as evidenced by the range of ICD-10-CM codes assigned to this subset of CCRs. Our subset of mitochondrial diseases therefore demonstrates how a collective understanding of the presentation of a specific condition may be best gained through comparisons with those of biologically similar diseases.

To validate the ICD-10-CM codes we assigned to each RMD report, we compare our code assignments to those expected for specific diseases. The list of diagnostic criteria identified for our set of six diseases generally follows consensus statements on mitochondrial disease diagnosis and treatment provided by Parikh *et al.*^28,^^29^ and the Mitochondrial Medicine Society (http://mitosoc.org/news/). Because these recommendations involve conditions that are frequently more specific than those described by ICD-10, we use this list primarily as a source of high-level types of symptoms. For example, acidosis (ICD-10-CM E87.2) is routinely described in each of the selected diseases, with the exception of Complex III deficiency. This symptom appears in 69 out of 246 reports, often as a specific form; some forms are represented by a unique ICD-10 code, such as 3-methylglutaconic aciduria (E71.111), while others, including lactic acidosis, rely on the general code for identification. Among the 5 mitochondrial respiratory chain complex deficiencies, none have a specific diagnostic code within ICD-10; we collectively annotate them with two codes for mitochondrial metabolism disorders (E88.40 and E88.49). Complex II and Complex V deficiencies are described in 12 and 7 reports in this subset, respectively, and are associated with novel hypertrophic cardiomyopathies (I42.2; seen in 7 reports across both disorders) and a lack of development in childhood (R62.50; seen in 7 reports across both). Complex III deficiency is associated with hypoglycemia (E16.2) in 17 out of 29 cases but is otherwise notable for its lack of distinguishing characteristics in this set. Generalized muscle weakness (M62.81), while common across all RMDs, is especially frequently described within reports on primary carnitine deficiency (18 out of 45, compared to 32 out of all 201 other reports). We also compare our code assignments to Barth syndrome diagnosis criteria; specifically, that the disease presentation frequently involves neutropenia and 3-methylglutaconic aciduria^30^. Out of 30 Barth syndrome reports, 26 describe neutropenia (D70.9), 24 describe dilated cardiomyopathy (I42.0), 16 describe cardiomegaly (I51.7), 11 describe acidosis (E87.2), and 8 describe 3-methylglutaconic aciduria (E71.111).

### Chronological features

The reports corresponding to metadata in the MACCR set were originally published between 1956 and 2018, with 455 CCRs published from 2017 to 2018. Out of all 3,100 reports contributing metadata to the MACCR set, 2,112 (68.1%) were published since 2007. Among 1.89 million CCRs indexed through PubMed/MEDLINE with publication dates from 1936 to August 2018, more than 595,000 (31.5%) were published since 2007. This skew toward more recent years in the MACCR set, relative to the distribution of all published CCRs, is partially a conscious choice made during curation to focus on cases with well-defined diagnoses and general similarity in terminology. This difference also reflects the greater accessibility of clinical case reports within the last decade.^1^ Two prominent sources are *British Medical Journal Case Reports* (*BMJ Case Rep*) and *Journal of Medical Case Reports* (*J Med Case Rep*), which began publishing in 2008 and 2007, respectively. These two journals alone have published more than 20,000 CCRs and contribute 166 reports (5.3%) to the MACCR set. Publications with longer histories, such as the *New England Journal of Medicine* (*NEJM*), continue to publish CCRs as well. Though their editorial stance on CCRs has changed over time^31^, *NEJM* has published nearly 10,000 case reports since 1949 and 2,224 since 2007, of which 101 and 75 are included in the MACCR set, respectively.

### Demographic and geographic distribution of MACCRs

The distribution of demographic features among patients described in our MACCR dataset shows it is not excessively skewed toward one subset of the patient population. Patient age presents a generally consistent distribution: 890 (28.7%) reports describe clinical presentations with pediatric patients (i.e., less than 21 years old), 1,104 (35.6%) reports describe patients of at least 21 and no more than 50 years old, and 1,051 (33.9%) reports describe patients of 51 years old or older. Just 55 (1.7%) reports did not provide enough information to know or infer patient age. Some reports may span years or decades of symptoms or treatment, so age remains a rough demographic value (in this case, age refers to patient age at the beginning of a given clinical narrative). Even so, we believe this is evidence that the MACCR set is not excessively biased in favor of a single age group.

Patient sex provides additional evidence of demographic and conceptual variety among CCRs contributing to the MACCR set. Over the 3,100 CCRs, 1,415 (45.6%) reports concern female patients, 1,536 (49.5%) concern male patients, and the remainder (149; 4.8%) did not specify a patient’s sex or were unclear. One potential source of imbalance between male and female patients is the presence of numerous cardiovascular disease (CVD) CCRs in our set: male patients with CVD symptoms are more likely to receive corresponding diagnostics and treatment *vs.* female patients^32,33^. Similarly, we find the assumption that all obstetrical and gynecological cases should involve female patients is generally true: out of 176 cases in this category, 160 identified female patients specifically; 14 reports did not explicitly state the patient’s gender, though it was implicitly inferred as female; and of the remaining 2 cases involving male patients, both describe conditions impacting male fetuses. Across all female patients, 360 are pediatric (<21), 551 were at least 21 and less than 51 years old, and 480 were 51 or older. Of the male patients, the same counts are 470, 513, and 530, respectively.

**Figure 3:**
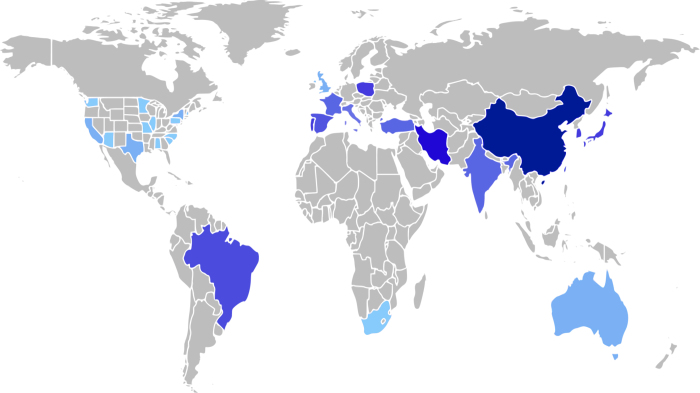
Geographic regions with difference in publication frequency. Here, we visualize the distribution of locations of reports in the MACCR set across the world and within US states. Darker shades correspond to a greater significant difference between the count of CCRs in the MACCR set for a particular region (determined by institutional affiliation of the corresponding author) and the count of all CCRs for that region (as specified by MEDLINE index).

This demographic variety extends to the geographic origin of the MACCRs: Fig. 3 provides a global view of differences between MACCR geographic origins and those of all CCRs. Across all CCRs published as of August 2018, more than 700,000 originate from the United States. Additional visualizations of the geographic distribution, including its breakdown by disease category, are shown in a supplementary animation (MACCR Supplementary File 1, Data Citation 1). Their distribution in this set is very similar to that seen across all MACCRs, even when accounting for individual states, though we note that location indexing is rarely comprehensive and provides only a rough estimate. Most reports in the MACCR set have corresponding authors from the US, Japan, and China, likely due to curation of reports from more recent years, and hence more publications from nations with higher citation rates in recent years^34^. As a result, China and Japan have a slightly greater representation in the MACCR set than in CCRs as a whole. It is important to note that these geographic identities relate to the corresponding author, which does not necessarily equate to the treatment location or origin of the patient. Patient geo-location data is not available from CCRs, though these data would be highly informative. Still, knowledge of the lead investigator’s geo-location provides crucial insight as to where important clinical work is occurring.

### Quality and value of case report metadata

The value of the MACCR dataset is derived from features created through metadata extraction. We recognize this is an *added* value, as the CCRs have existing metadata and subject headings provided by MEDLINE and MeSH (e.g., the content of each title, author list, and other bibliographic information). Unlike MeSH assignments available through PubMed, we have examined the full clinical narrative communicated within each case report and therefore furnish more detail than that permitted by the short list of MeSH descriptors associated with each document. Our inclusion of descriptions of clinical events and activities as they are described in the case reports themselves covers terminology not present in MeSH, particularly for the names of drugs and diagnostic or therapeutic procedures. Our metadata extraction approach also differs from a code-centric approach and from NER-driven text mining approaches in that it relies upon assignment of document text to general clinical concepts rather than to stringently-defined concepts or entities. Neither MeSH nor ICD-10-CM have been designed with clinical narratives in mind: the former is intended to index biomedical documents while the latter translates clinical diagnoses into coded endpoints. Our metadata extraction approach fills the niche these methods were not intended to address.

As part of our metadata extraction process, we used a scoring system to quantify the total number of features manually identified across all MACCRs. A CCR with a full set of values corresponding to clinical concepts (i.e., each of the concepts has at least one associated text value) is assigned a medical content score of 18, the highest value. One point each is provided for presence of key words and for enough detail to determine a disease category, while the remaining points reflect types of medical content. The minimum medical content score is 1. The average medical content score across all metadata sets is 10.9 with a standard deviation of +/−3.52, indicating each set of metadata provides more than 10 new details on average, relative to available metadata, or more than 33,000 new details across all metadata records in the set. More than 7,400 details are from CCRs discussing rare disease presentations. It is relevant to note that the presence of text values corresponding to each medical concept is not consistent across all concepts and across the full MACCR set; we believe this indicates material was simply not included in the CCRs rather than omitted during the metadata extraction process. For example, 2,980 (96%) of the MACCRs contain material describing diagnostic techniques and procedures, 2,349 (75.7%) contain material describing clinical outcomes, and just 186 (6%) provide descriptions of patient social history.

We may also consider the metadata value in terms of information entropy. Determining entropy on a per-character basis (i.e., Shannon entropy) allows us to calculate an average entropy per concept, with all “NA” values treated as a value with an entropy of 0 bits (as these values provide no additional information). We use this metric in lieu of estimates of readability (e.g., Flesch-Kincaid^35^ or SMOG^36^ scores) as these metrics are heavily biased by the frequency of complex vocabulary common to medical language. We intend the entropy values to serve as estimates of differences in information content between fields in the main MACCR dataset. Table 4 contains these average entropy values, along with character, word, and segment (i.e., each semicolon-delimited phrase) counts for each medical concept. This approach essentially combines two metrics (i.e., average entropy for a measurement of overall information content and treatment of “NA” values to adjust for missing values) such that the resulting entropy values denote estimates of each concept’s overall semantic complexity relative to others. Concepts with average entropy close to 4, such as Diagnostic Techniques and Procedures, not only contain more values than other concepts but each value is more complex, primarily as a function of length.

## Usage Notes

We anticipate the MACCR set will aid clinicians and clinical researchers in gaining a better understanding of disease presentations, including their key symptomology, diagnostic approaches, and treatment. Researchers in bioinformatics, clinical informatics, and information extraction will find the MACCRs useful as a set of medical language labeled at multiple levels. Individual researchers faced with small sample sizes may use the MACCRs to enhance their statistical power through incorporation of additional observations, or as a starting point for the assembly of *in silico* patient cohorts. We envision a researcher could generate MACCRs of their own using our metadata standard template to assemble these cohorts and leverage the cumulative power for statistical analysis. The resulting metadata enables deep text mining alongside MeSH, ICD-10, and other clinical ontologies. To establish the utility of the dataset and guide those interested in employing it in their research, we present use cases that may be pursued by researchers, physician investigators, clinicians, data scientists, IP officers, pharmaceutical companies for drug development, and those shaping government policies for clinical trials. This set of metadata in medical language yields a rich resource for providing insight into the events and biological phenomena within each clinical presentation.

### Analysis of case report features

The immediately applicable uses of the MACCR set are those involving aggregation and analysis of features particular to each CCR. As the reports contributing metadata to this set reflect a substantial variety of disease presentations and features, our structured data provide a multitude of options for subsequent analysis. In the simplest case, extraction of sets of terms associated with a particular disease or disease category establishes a set of starter terms for further study. For example, extraction of all diagnostic procedures used in respiratory disease cases in the MACCR set (e.g., chest x-ray, lung function test, or CT scan) allows researchers to better direct future literature searches by including a set of commonly used treatments. These terms, while not comprehensive for any particular topic, form a representative set of term *vs.* concept associations and include nonspecific terms as well (e.g., CT scans are not specific to diagnostics for respiratory tract disease).

The rich term vocabulary available within the MACCR set permits more in-depth analysis and application to additional documents. Researchers may find this metadata particularly helpful in studying differences in treatment approaches across disease type or on the basis of the impacted organ system. We suggest that an initial analysis of this set be managed through extraction of a set of terms in a comprehensive dictionary, such as RxNorm^37^. Starting with observations listed in the Drug Therapy column in each metadata record, for example, rule-based and NER methods can identify compound names of interest. Enrichment of any name or group of names among a given subset of the metadata will reveal broader phenomena, e.g., antibiotics may correlate with infectious disease cases.

Because the MACCR set includes English medical language from a variety of locales, it is a representative set of medical text not specific to a single region, organization, or population. The dataset includes structured demographic features (including age, sex, and geo-location) to serve as easily-parsed features for correlative analyses. A more in-depth search enables geography-based analyses: by combining both document metadata and free-text from metadata categories (specifically, patient demographics or other features describing the patient), these fields can be mined for names of major cities, states, and countries. They may then be mapped and quantified to visualize the case report distribution (as demonstrated in Fig. 3). Methodologies developed using this dataset are appropriate for multi-level geographic term identification (i.e., from specific to general location) with a larger set of clinical reports, especially as researchers may find that certain features of CCRs are more informative for geographic location than others. Geographic trends revealed using the MACCR set may reveal broader phenomena to be followed up in new studies. They may also provide evidence of an unexpected focus in regional publication for specific diseases. We may expect CCRs involving a regional epidemic of a specific infectious disease to be predominantly written by clinicians in that area. Alternatively, these cases may also be popular among clinicians describing the spread of infectious disease to new locations.

Incorporating CCR analysis into broader studies permits exploration of undefined or poorly defined diagnoses. Some rare diseases may only exist, conceptually, as subsets of more common disease presentations. Heart failure, for example, is such a common condition that it may be responsible for more hospitalizations than any other condition, yet half of heart failure patients may suffer from a particular subtype of the syndrome, heart failure with preserved ejection fraction^38^, or HFpEF. Despite its current prevalence, HFpEF was only recognized as a distinct condition within the last several decades. The first observations of disease presentations lacking specific diagnostic consensus may therefore be contained only in CCRs and are unlikely to appear in formal epidemiological studies.

### Support for mitochondrial and rare disease characterization

The MACCR dataset documented here contains a rare disease subset, including a set of rare mitochondrial diseases. There are over 7,000 rare diseases affecting over 300 million individuals worldwide, 58 of which are RMDs (https://rarediseases.info.nih.gov/diseases). We have generated metadata acquired from CCRs describing 7 mitochondrial diseases for this dataset, as well as an additional set of ICD-10-CM diagnostic and symptom codes. The RMDs curated for this dataset have a limited number of publications in the medical literature, so our metadata collectively represent a substantial portion of the published clinical observations of these diseases. Each of the RMDs shares similar features in that they all involve mitochondrial abnormalities, yet each produces markedly different phenotypes and clinical signs. A number of analysis routes are available with the current dataset. We suggest that researchers use this set as a model to identify additional cases in the literature not explicitly identified as mitochondrial diseases. These implicit cases may be more common than anticipated but may be predicted based on presentations sharing numerous signs and symptoms with known cases. The resulting predicted cases would comprise a model for future studies of rare and/or idiopathic disease. For RMDs in particular, the MACCR set establishes a basis for both expected and frequently correlated symptomology, facilitated by the ICD-10-CM on RMD CCR symptomology.

Additional data on the genetic and molecular basis of the selected RMDs provides a more specific diagnostic picture and allows researchers to interface with knowledgebases to conduct proteome and pathway analysis. This information can offer mechanistic insight for the potential pathogenesis of disease. The etiology of many RMDs lies in mutations to mitochondrial DNA (mtDNA), but the majority are caused by mutations to nuclear-encoded genes that play various critical roles in mitochondrial biosynthesis and function (Fig. 2c). Barth syndrome, for instance, is caused by mutations to the *TAZ* (*G4.5*) gene at Xq28, resulting in malformed mitochondrial membranes due to a nonfunctional tafazzin enzyme that is responsible for adding linoleic acid to cardiolipin (CL) through its acyltransferase activity. Because of its prominence in the mitochondrial inner membrane and its intimate association with the electron transport chain complexes, improperly formed CL severely limits mitochondrial energy production and results in a variety of complications, including dilated cardiomyopathy, hypertrophic dilated cardiomyopathy, left-ventricular non-compaction, and endocardial fibroelastosis. Barth syndrome was first described in 1983^30,39^ and over 100 distinct mutations to the *TAZ* gene have been identified since the genetic basis was discovered in 1996^40^. Recognizing the common feature of diminished CL and elevated concentrations of monolysocardiolipin (MLCL) in Barth syndrome, an HPLC-MS/MS bloodspot assay was developed for diagnosis by an elevated MLCL:CL ratio^41^. The MACCR set can serve as a valuable resource for uncovering common proteins, pathways, and metabolites of interest in RMDs, which may lead to the identification of potential biomarkers. The depth of information can be further amplified by integrating this dataset with other publicly available resources and knowledgebases such as *UniProt*^42^ (http://www.uniprot.org), *Reactome*^43,44^ (https://reactome.org), and the Human Metabolome Database (HMDB)^45^ (http://www.hmdb.ca).

This dataset will aid users in gaining a better understanding of rare diseases and their treatments through downstream analysis of the structured text data. The CCR metadata derived from reports on Barth syndrome, for example, are directly relevant to developing and evaluating treatments for this and related conditions, as well as diagnostic and treatment planning in the clinic. By reviewing existing CCR metadata, a researcher could investigate past treatment regimens to analyze recorded symptoms or side effects and the degree of improvement under prevailing standards of care. Similarly, clinicians could employ metadata extracted from CCRs to support differential diagnosis and treatment planning in a patient with a suspected mitochondrial disorder. Furthermore, we envision that researchers and clinicians might utilize the metadata standard template and protocols to generate additional MACCRs and construct *in silico* patient cohorts of their own, enhancing their ability to compare existing treatments and evaluate newly developed therapies. For example, Elamipretide (Stealth Biotherapeutics, Newton, Massachusetts) is currently under stage II and III clinical trials for treatment of Barth syndrome and other mitochondrial myopathies by protecting properly formed cardiolipin from damage^46^. As new case reports on patients treated with Elamipretide become available, additional MACCRs might be leveraged to compare treatments, perhaps identifying side effects or varying efficacy of the new drug in different patient groups.

### Support for congenital heart disease characterization

Congenital heart defects (CHD) and disease are unfortunately common clinical issues occurring in an estimated 1% of births in the United States^47^. Similarly, they are frequently represented among the reports in the MACCR dataset, 243 of which involve CHD. Though general descriptions of CHD diagnoses (e.g., “ventricular septal defect” or “hypoplastic left heart syndrome”) lend themselves well to indexing by MeSH, more nuanced narratives surrounding CHD presentations require more detailed examination. As a brief example, among the CHD reports within the MACCR set, the top MeSH descriptors include “Infant, Newborn”, “Echocardiography”, and “Abnormalities, Multiple”. A comparison of the metadata for these reports reveals some variation in age: of the 240 CCRs for which age is specified or can be inferred, mean age is about 22.7 years, with 76 cases involving patients less than a year old and 137 involving pediatric patients (any under 21 years of age). In terms of additional detail, however, just 49 (20.2%) of these CCRs specifically mention heart failure, but nearly as many (47) mention cyanosis or cyanotic conditions. Though a larger collection of CHD reports may be required, metadata extraction and investigation of these reports may provide solid evidence for new biomarker candidates in an area where few currently exist.

### Models of medical language

The MACCR set contains rich, contextual descriptions of medical events. Individual words and phrases in the set are not explicitly assigned to a given ontology or vocabulary but are included within our medical concept categories. For example, instead of indicating a document describes a “myocardial infarction” and/or identifying this phrase in each document, if a document mentions events such as a heart attack, we assign the event to the appropriate medical concept (and assign a disease category; in this case, cardiovascular disease). The corresponding text segment in each case includes phrases such as “family history was positive for myocardial infarction in a sibling at age 54 years”^48^. As compared with an approach of processing unstructured case report text with NLP tools, our resource supplies an intermediate level of structure sufficient to retain the context of the segment. This semantic context contains information denoting relations between concepts and events with enough detail to assign additional diagnostic categories and codes. In the example of a myocardial infarction, the metadata often includes the details necessary to determine subtypes (e.g., ST-elevation *vs.* non-ST elevation, or ICD-10-CM codes of I21.3 *vs.* I21.4). Employing NER alone on the source text may yield only phrases such as “myocardial infarction”, and MeSH descriptors generally do not index documents to this level of detail. The MACCR set labels text segments with medical concepts, allowing collections of phrases, rather than named entities, to be associated with higher-level concepts. Our resource thereby enables an additional degree of interoperability between CCRs, controlled vocabularies (e.g., MeSH), and diagnostic coding systems (e.g., ICD-10), while supplying a rich collection of contextual and concept-labeled clinical text features.

The contents of the MACCR set provide the structured training data necessary for developing computational models of higher-level features in clinical text. Computational linguists as well as researchers in clinical informatics and medical NLP may use the MACCR records to develop concept-level models of medical language, allowing for context-based machine learning and alternatives to dictionary-driven NLP approaches^49,50^. The MACCR approach supports generation of term frequency sets, word vectors, and basic entity-level analysis (e.g., with UMLS resources^25^) while retaining clinical concepts such as medical history or demographics. NER systems such as cTAKES^49^ (http://ctakes.apache.org/) or CLAMP^50^ (http://clamp.uth.edu/) support identification of procedures and signs/symptoms using the features within MACCR records. Additionally, NER and rule-based phrase matching approaches draw connections between MACCR content and biomedical knowledgebases (i.e., *UniProt*^42^ [http://www.uniprot.org], *Reactome*^43,44^ [https://reactome.org], or the Disease Ontology^51^ [http://disease-ontology.org/]). The combination of extensive knowledgebases with advanced computational models supports transformation of clinical observations into biomedical insight.

Our dataset facilitates expansion of ongoing developments in NLP principles and methods to medical documents, especially alongside the substantial extant resources for contextualization and distant supervision of computational approaches to understanding medical language. The broad demand from the community and far-reaching significance of NLP approaches has been a springboard for novel approaches in biomedical research and beyond, evidenced by rapid development of tools and resources in a variety of research fields^52–55^. Beyond clinical informatics research, tools developed to enforce structure on otherwise unstructured biomedical text – including that in electronic health records – offer a major source of untapped biomedical knowledge^56,57^. These tools will require significant software development and engineering efforts, yet once the resulting knowledge becomes structured and searchable, it will be of greater interest and utility to clinicians, data scientists, and physician investigators, as well as intellectual property specialists and officials determining policies for clinical trials.

Manual curation is currently the most suitable option for capturing comprehensive details associated with high-level concepts in biomedical literature. Though it may someday yield similar results, automated, machine learning-driven medical language analysis presents distinct limitations in precision and recall, producing numerous false positive and false negative results as compared to human annotators^58^. With this dataset, we present a resource appropriate for training new machine learning models on the types of language common to clinical case reports: vocabulary, common phrases, and association with high-level medical concepts. The resulting models may then support further human curation and metadata extraction, assembly of more fine-grained knowledge structures (e.g., knowledge graphs), and transfer learning to train more complex medical language models. Our dataset is therefore entirely complementary to biomedical text resources such CRAFT^15^ and those available through i2b2^13,14^. Though these datasets provide records of specific concepts and features, the MACCR set furnishes rich metadata of clinical concepts across a wide variety of disease types. In instances where machine learning methods may require considerably a larger amount of text for training, we suggest using the MACCR set as an initial training step and in combination with other text resources.

### An educational resource for writing better case reports

The MACCR set contains metadata for reports spanning disease types and medical specialties, highlighting a wide variety of CCR writing styles and a range of completeness in describing relevant clinical concepts. In some cases, variance among MACCRs is the result of a lack of explicitly stated observations: e.g. a patient’s exact age or family history may be omitted. Similarly, clinicians may not mention tests if the diagnostics or their results were considered trivial. The richness of our dataset offers a basis for comparison among cases. Clinical investigators may observe the extent to which expected clinical concepts are or are not discussed in case reports. This analysis may be particularly informative if otherwise similar cases ares found to differ in diagnosis. Other features may be more useful for subsequent analyses if provided in a more specific, quantifiable manner; a CCR with a patient described as “55 years old”, for instance, will be more informative than one with the description “middle-aged.” Further analysis of the specific features within CCRs will provide clear examples of how clinicians may write more informative, citable, and computationally readable CCRs.

While, at present, case reports are primarily read by academic physicians for educational purposes, implementation of the standardized metadata template to enrich these documents can expand the audience and application of case reports. For example, case report user groups may include medical students, interns and fellows, epidemiologists, and statisticians. These audiences would not only be able to more easily identify relevant CCRs, but also derive valuable information from improved indexing and categorization. In turn, these improvements will lead to better understanding of clinical phenotypes and relationships of an individual case to a larger representative patient population. As another example, healthcare organizations and policymakers (e.g., FDA) can retrieve CCR metadata as an additional source for tracking unusual disease occurrences, epidemiological trends, and post-marketing drug surveillance. Moreover, pharmaceutical industries can design a survey on case reports of drugs with unexpected indications or unrecognized side-effects to assist in modifying usage instructions and direct future development.

To address the key clinical items commonly missing in case reports, we envision a solution that integrates what PubMed has already accomplished with MeSH terms using both metadata extraction and coding with ICD-10-CM. This strategy would support further classification with systems such as the International Classification of Health Interventions (ICHI) (http://www.who.int/classifications/ichi/en/) to compensate for missing items. The resulting curated, indexed, and structured CCR metadata could ultimately interface with preclinical -omics research, clinical cohort studies, and clinical trials to advance understanding of disease progression, management, and clinical outcomes. To surmount the ever-growing amount of free-text information with limited metadata, indexing, and accessibility, computational platforms and in-depth search algorithms will enable better recognition of CCR contents and relevant clinical trials to elevate text data analysis, advance medical science, and improve patient care.

As a time-honored tradition in medical publication and a treasured source of clinical data, clinical case reports augment our understanding of disease etiology, pathogenesis, miscellaneous diagnosis, and therapeutic efficacy. These reports provide valuable clinical narratives relevant to clinicians and biomedical researchers. The growing volume of case reports published each year stands testament to their popularity and usefulness to their targeted clinical readership, but this size, coupled with the isolated, unstructured, and heterogeneous nature of case reports’ contents, also presents a challenge to index, annotate, and query case report data. In this report, we created a standardized metadata template and metrics, as well as a test dataset consisting of 3,100 CCRs spanning 16 disease categories. In the course of assembling our dataset, we evaluated the caliber of the existing metadata employed for case reports in PubMed and confirmed a discrepancy between the medical content and the metadata meant to describe it. Our MACCR set addresses this discrepancy by adding rich metadata and serves as a valuable resource for biomedical researchers developing novel approaches to advance medical science and improve patient care^20^.

## Additional information

**How to cite this article**: Caufield, J. H. *et al*. A reference set of curated biomedical data and metadata from clinical case reports. *Sci. Data*. 5:180258 doi: 10.1038/sdata.2018.258 (2018).

**Publisher’s note**: Springer Nature remains neutral with regard to jurisdictional claims in published maps and institutional affiliations.

## Supplementary Material



## Figures and Tables

**Table 1:** Standardized metadata template for clinical case reports.

Field	Data Type	Example
**Case Report Identification (Findable)**		
Title	Text	Case report: a case of cardiogenic shock and hyperparathyroidism.
Authors	Text	Neeley AB, Mossman ET
Year	Text	2017
Journal	Text	Midwest Journal of Medicine
Institution	Text	Department of Cardiology, Mt Vernon Hospital, Mt Vernon, Wisconsin, USA
Corresponding Author*	Text	Neeley AB
PMID	Identifier	29999555
DOI	Identifier	10.1011/mwjmed.2017.10.001
Link	Identifier	http://www.mwjmed.org/doi/full/10.1011/mwjmed.2017.10.001
Language*	Text	English
**Medical Content (Accessible, Interoperable, Reusable)**		
Key Words	Text	Shock, cardiogenic; hyperparathyroidism; fatigue; headache
Demography^**^	Text	Male; 40 years of age
Geographic Locations^***^	Text	Mt Vernon, Wisconsin, USA
Life Style	Text	Smoker
Family History	Text	no family history of heart disease
Social History	Text	worked as a truck driver
Medical/Surgical History	Text	history of fatigue; splenectomy performed six years previously
Disease System	Text	Cardiovascular diseases
Signs and Symptoms	Text	presented with lethargy, headache, diaphoresis, and twitching in all four limbs; cardiac enzyme levels were elevated, ventricular tachycardia
Comorbidity	Text	alopecia
Diagnostic Techniques and Procedures	Text	Electrocardiogram; dual energy X-ray absorptiometry (DXA)
Diagnosis	Text	hyperparathyroidism
Laboratory Values	Text	serum calcium concentration was 3.0 mmol per liter; complete blood cell counts normal
Pathology	Text	endomyocardial biopsy did not reveal a myocardial pathology
Pharmaceutical Therapy	Text	bisphophonates
Interventional Therapy	Text	ventilated on the 2^nd^ day post-surgery due to respiratory distress
Patient Outcome Assessment	Text	Patient developed refractory shock; died of persistent ventricular tachycardia
Diagnostic Imaging/Videotape Recording^****^	Numerical	3;0;0;0
Relationship to Other Case Reports*	Text / Identifier	PMID: 5555555
Relationship with Clinical Trial*	Text / Identifier	PMID: 5551111
Crosslink with Database*	Text / Identifier	MedlinePlus Health Information : https://medlineplus.gov/parathyroiddisorders.html
**Acknowledgements**		
Funding Source	Text	National Institutes of Health/National Heart, Lung, and Blood Institute
Award Number	Identifier	R01HL123123 (to AN)
Disclosures/Conflict of Interest	Text	Dr. Neeley is a paid consultant for Medicaltech Inc.
References	Numerical	12
A set of features common to clinical case reports and facilitating their concept-level metadata extraction. This template is arranged into three primary sections: Identification, Medical Content, and Acknowledgments, denoting the purpose and additional value afforded by each type of case report feature. Here, we have also included relevancy of the first two categories to promoting FAIR standards. A single document contains the majority of these features; metadata records include the span of these features (i.e., the value referring to a single concept). Examples shown here are simulated but representative of dataset contents. Data Type refers to the type of source data, rather than the dataset contents; this may be “Text” if free-text (this may contain numerical components, though these are identified in subsequent processing steps), “Identifier” if a unique database identifier or other structured value specific to the document, or “Numerical”. Please note that the Acknowledgements section provided here, including the Disclosures/Conflict of Interest statement, is an example only and not intended to claim any funding provided to or competing interest by the authors.		
^∗^The template and associated processing workflow support use of these fields, though their values are not provided in the MACCR set.		
^∗∗^Demography details are converted to consistent values prior to inclusion in the MACCR set.		
^∗∗∗^If not provided within document text, geographic location is inferred from the associated institution.		
^∗∗∗∗^Purely a numerical count of the total number of clinical images, figures, videos, and tables, respectively, published along with the main text of the report.		

**Table 2:** Disease system categories.

Category	Description
cancer	cancer or neoplasms
nervous	brain, spine, or nerve involvement
cardiovascular	heart or cardiovascular involvement, not including conditions specific to the blood
musculoskeletal and rheumatic	muscle, bone, joints, or connective tissue involvement
digestive	gastrointestinal involvement, including liver, pancreas, or gallbladder
obstetrical and gynecological	pregnancy, childbirth, the female reproductive system, or the breasts
infectious	infection by microorganisms
respiratory	respiratory tract involvement
hematologic	blood, bone marrow, lymph nodes, or spleen involvement
kidney and urologic	kidney or bladder involvement; any involvement of the male reproductive organs
endocrine	endocrine gland involvement and metabolic disorders
oral and maxillofacial	mouth, jaws, head, face, or neck, including dental issues
eye	eye involvement and visual issues
otorhinolaryngologic	ear, nose, or throat involvement, including hearing issues
skin	skin involvement
rare	rare diseases; see above
A set of categories for grouping case reports on the basis of related symptoms, co-morbidities, and etiologies. A report may belong to more than one category, particularly in clinical presentations involving multiple disease systems and/or cancer of one or more systems. The special category of ‘rare’ is specific to reports of rare diseases affecting no more than 200,000 individuals in the United States at a time.	

**Table 3:** Support for FAIRShake metrics.

FAIRshake metric	Score	Support for metric
1. A standardized ID or accession number is used to identify the dataset.	Yes; 1	Dataset provided with unique DOIs by Figshare and Dryad.
2. The dataset is described with metadata using a formal, broadly applicable vocabulary.	Yes; 1	Dataset is described using formal biomedical terminology, including diagnostic techniques and procedures, signs and symptoms, etc., as well as MeSH terms and ICD-10 codes.
3. Information is provided on the experimental methods used to generate the data.	Yes; 1	All methods for ACCR metadata template generation provided along with raw data files.
4. The dataset is hosted in an established data repository, if a relevant repository exists.	Yes; 1	Dataset is hosted on the Figshare and Dryad repositories.
5. The dataset can be downloaded for free from the repository.	Yes; 1	Dataset can be downloaded in .tsv format for free from the Figshare and Dryad repositories.
6. Version information is provided for the dataset.	Yes; 1	Versioning begins with v1, and updated versions of the set will be v2, v3 etc.
7. Contact information is provided for the creator(s) of the dataset.	Yes; 1	Contact information for the lead investigator is provided (Dr. Peipei Ping).
8. Information is provided describing how to cite the dataset.	Yes; 1	Citation information is provided along with this publication.
9. Licensing information is provided on the dataset’s landing page.	Yes; 1	Licensing information is provided through Figshare, Dryad, and *Scientific Data*.
Here, we provide scores for each of the metrics in the FAIRshake (https://fairshake.cloud) rating system. The MACCR dataset fully meets each metric and therefore has a score of “1” for each.		

**Table 4:** Text properties and entropy of medical concept metadata records.

Concept	Average Entropy (bits, +/−standard deviation)	Character Count	Word Count	Segment Count
Keywords	2.17 +/− 2.04	127,932	8,326	6,636
Geographic Locations	0.35 +/− 1.01	6,085	901	358
Life Style	0.55 +/− 1.35	29,244	4,862	521
Family History	1.15 +/− 1.83	138,162	21,342	1,717
Social History	0.23 +/− 0.90	12,310	2,022	249
Medical/Surgical History	3.02 +/− 1.84	804,975	119,816	8,783
Signs and Symptoms	3.96 +/− 0.94	1,460,450	218,276	16,467
Comorbidities	0.96 +/− 1.63	33,978	3,918	1,329
Diagnostic Techniques and Procedures	3.98 + /− 0.87	1,369,668	195,000	15,936
Diagnosis	3.85 +/− 0.66	206,418	24,432	4,718
Laboratory Values	2.80 +/− 2.12	990,769	146,240	5,238
Pathology	2.32 +/− 2.11	853,084	121,009	2,865
Pharmacological Therapy	2.74 +/− 1.99	422,402	60,270	3,863
Interventional Therapy	2.60 +/− 1.94	399,831	57,967	4,909
Patient Outcome Assessment	3.07 +/− 1.77	440,602	66,786	4,526
For each medical concept used in the metadata extraction process, we determined its average character-level entropy (Shannon entropy) across all text values in the concept, along with its standard deviation. As length of text can contribute to estimates of its complexity, we also include counts of characters (not including delimiters or spaces), words, and segments (i.e., phrases between delimiters) for each concept across the MACCR set. Values of “NA” are considered to have an entropy of zero and do not contribute to character, word, or segment counts.				
